# Dr. Samuel S. Kilvington

**Published:** 1911-11

**Authors:** 


Dr. Samuel S. Kilvington, University of Buffalo, 1883, died at Minnetonka Mills, Minn., of cerebral hemorrhage, on September 25, 1911, aged 69.

